# An App-Based Remote Patient Monitoring System With Wrist and In-Ear Wearables in Gastrointestinal Oncology: Prospective Feasibility Pilot Study

**DOI:** 10.2196/64184

**Published:** 2025-10-30

**Authors:** Lara Kohn, Veit Scheble, Philip Storz, Anita Müller, Selcan Behiye Ulas, Fee Schmitt, Christian Thies, Nisar Malek

**Affiliations:** 1 Center for Personalized Medicine, University Hospital Tübingen, Röntgenweg 9, Tübingen, 72076, Germany, 49 7071 29 82864, 49 7071 29 2727; 2 Clinic of Internal Medicine, University Hospital Tübingen, Tübingen, Germany; 3 Reutlingen Research Institute, Reutlingen University, Reutlingen, Germany; 4 M3 Research Center, University of Tübingen, Tübingen, Germany

**Keywords:** feasibility, gastrointestinal, remote patient monitoring, app, acceptability, cancer, precision medicine, pilot, mobile health (mHealth), wearables

## Abstract

**Background:**

Outpatient treatments, including targeted therapies, in oncology are on the rise. The implementation of remote patient monitoring (RPM) between therapy sessions has the potential to enhance patient care and therapy outcomes in the future.

**Objective:**

This pilot study assessed the feasibility of a new, app-based RPM system (bwHealthApp) by capturing patient-reported outcomes (PROs) and vital parameters using a wrist- or in-ear wearable. The study examined adherence, acceptance, and satisfaction, as well as the differences between the two types of wearables.

**Methods:**

Outpatients with gastrointestinal cancer receiving systemic therapies were invited to use and evaluate the bwHealthApp system for 1 month. The system was set up as an Android smartphone app to assess electronic PROs (ePROs) and connect to wearables for continuous vital sign measurements. A wrist wearable (Beurer AS99) that measured activity and heart rate or an in-ear wearable (Cosinuss Two) that measured heart rate, oxygen saturation, and temperature was used. The data were synchronized remotely to a web client. Outpatients were randomly assigned to the wrist wearable (n=17) or in-ear wearable (n=14) group. At the beginning, middle, and end of the study, patients (N=31) completed questionnaires on various feasibility aspects. Adherence to ePRO completion; wearable use, acceptance, and satisfaction; wearable data quantity and quality; and differences between the wearables were evaluated.

**Results:**

The mean adherence to bwHealthApp including dropouts was 46% (449/971 days) for ePRO completion and 61% (593/971 days) for wearable use. The system was most frequently used during everyday activities. More than half of the participants were satisfied with bwHealthApp (18/30, 60%) and could imagine continued use (21/30, 70%). Notably, 70% (21/30) rated the system as easy to use. Participants recorded more than 20 million wearable measures; however, 29% (SD 22%, range 3%-76%) of temperature values and 33% (SD 25%, range 10%-92%) of oxygen saturation values were outside the physiological range. For heart rate, the mean proportion of excluded values was 10% (SD 11%, range 4%-48%; in-ear) and 11% (SD 8%, range 4%-33%; wrist). The frequency of use of the two wearables did not differ significantly (*t*_29_=1.81; *P*=.08). The wrist wearable scored significantly better than the in-ear wearable regarding wearing comfort (*t*_28_=−11.17; *P*=.03).

**Conclusions:**

This bwHealthApp pilot study demonstrates the feasibility of RPM via a mobile app and wearable devices during outpatient systemic cancer therapy, including targeted therapies. The adherence level was moderate, and patients were generally satisfied with the system, although the wrist wearable received a higher rating. The system’s functionality may be enhanced through the integration of additional wearables. This pilot study serves as a foundational framework for long-term assessments examining RPM and clinical data to improve cancer treatments.

## Introduction

Systemic cancer therapies are frequently used in the outpatient setting. Personalized biomarker-based treatment strategies lead to increasingly individualized therapy regimens, which pose new challenges for patient monitoring, especially if therapy sessions are several weeks apart.

Missing monitoring may lead to late detection of treatment failure, adverse drug effects, and worsening of an outpatient’s condition. In addition, recall bias may result in incorrect patient information. Therefore, subjective assessments should be supplemented by objective measures of vital parameters that show associations with the treatment.

The resulting monitoring gap and bias can be addressed by remote patient monitoring (RPM), in which patient assessments are transmitted electronically, for example, via mobile apps. Objective sensor measurements complement the subjective data and provide important real-world data (RWD). RWD are derived from various sources, such as electronic patient-reported outcomes (ePROs) and sensor measurements [1], and are associated with outcomes in heterogeneous outpatient populations in real-world settings. In precision oncology cohorts, RWD play a pivotal role and represent a data repository for future improvement of patient care and therapy by detecting serious side effects as well as potential therapy failure at an early stage [2].

Precision medicine has an important impact on outpatient treatment regimens for prevalent disease entities such as gastrointestinal cancers [3]. For example, adenocarcinomas of the stomach and esophagogastric junction are treated with the checkpoint inhibitor nivolumab [4]. Unlike conventional chemotherapeutic agents, precision medicine drugs target the molecular structure of the tumor. Immunotherapy may lead to immune-related adverse events, such as autoimmune pneumonitis, which can be detected by monitoring oxygen saturation and ePROs (such as dyspnea) [5]. Other parameters are also important for monitoring purposes; for example, body temperature can be used to detect chemotherapy-induced neutropenia.

Monitoring requires technology. Although both smartphones and fitness trackers are well established in society, it remains uncertain whether their use in the medical field will be accepted. Currently, there are no guidelines on RPM in the context of personalized cancer therapy. Previous studies show that physical activity is the most frequently examined factor. Physical activity can be increased by wearing fitness trackers and is associated with improved quality of life and lower mortality [6,7]. Physically inactive patients are more likely to be hospitalized [8]. Thus, measuring physical activity is important for disease and treatment monitoring. Physical activity is typically measured with wearable wristbands, which have been the focus in previous RPM studies on patients with cancer [9,10]. Other types of wearables such as in-ear sensors have not been assessed in this context. These offer the advantage that the body temperature and oxygen saturation are continuously recorded. However, potential differences in the use and acceptance of the two wearable systems (wrist and in-ear), especially in critically ill patients, have not yet been examined.

Numerous nonmedical wearable devices are available on the market. It has not been established whether they can be used to obtain clinically usable data that are correlated with cancer progression indicators such as laboratory results or staging. In future, these could be used to detect, for example, early signs of a condition worsening, which could be validated in the clinic. The vision is that patients will connect personal wearables to a monitoring system and provide low-threshold data for monitoring. This approach is expected to improve adherence and economic outcomes in the future and enable rapid adaptation to technical innovations in wearables.

For this purpose, a new RPM system—bwHealthApp—was created with ePROs and connected commercial wrist and in-ear wearables. Prior to analyzing correlations with clinical progression indicators, a pilot feasibility study was conducted. This pilot study aimed to test bwHealthApp for feasibility in patients with late-stage cancer. Feasibility in this context focuses on acceptability, adherence, use rates, and satisfaction. Moreover, the study aimed to analyze the differences in use and satisfaction between a wrist wearable and an in-ear wearable.

## Methods

### Design

**Figure 1.**
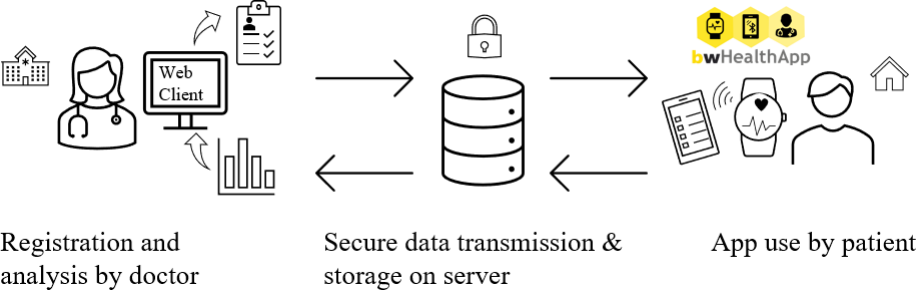
Study procedure for using the bwHealthApp software platform with the web client.

This study was a prospective feasibility pilot study using bwHealthApp to record information on health status (via ePROs), vital and activity parameters via wearables worn on the wrist or in the ear (Figure 1). The study period involved 3-5 systemic therapy cycles, leading to a duration of 3-8 weeks, depending on the therapy regimen. Surveys on bwHealthApp were conducted over 3 visits at the hospital. The outcomes were descriptive adherence to ePRO completion and wearable use as well as self-reported (technology) acceptance of and satisfaction with the bwHealthApp system. Participants were randomly assigned to the wrist or in-ear wearable. The outcomes were the descriptive quality of sensor measurements and significance of the difference in use variables and satisfaction.

### Participants

Thirty-two outpatients of a gastrointestinal-oncological unit at the University Hospital Tübingen were recruited in consultation with their oncologist between July and October 2022. The sample size was calculated for the feasibility analysis. Due to the variation in wearable type and the first-time use of the system, it was expected that the differences between the self-perceived use rates at the beginning and end of the study between the two types of wearables would be medium (partial η^2^≥0.06; n=34) to strong (partial η^2^≥0.14; n=16) using repeated measures ANOVA (power 0.8; α=.05; calculated using G*Power [11]). A sample size of 34 was considered sufficient. During the planned recruitment period, 32 patients were willing to participate. Women and men older than 18 years with a histologically confirmed gastrointestinal malignancy and receiving systemic therapy were included in the study. The exclusion criteria were preexisting diseases associated with restricted mobility and preexisting conditions that make it unfeasible to operate a smartphone app. First, 100 eligible patients were informed about the study by an MD student. Patients interested in participating received an information flyer. At the next treatment session, the patients stated whether they agreed to participate. After informed consent was obtained, participants were randomly assigned to two groups by drawing a paper lot marked with a group code: the wrist wearable group (Beurer AS99; n=18) or in-ear wearable group (Cosinuss Two; n=14). In total, 20 participants used the app on their own Android smartphone, and 12 were provided with a smartphone (Samsung A70) due to the absence of an iOS version of the app at the time.

### Procedure

**Figure 2.**
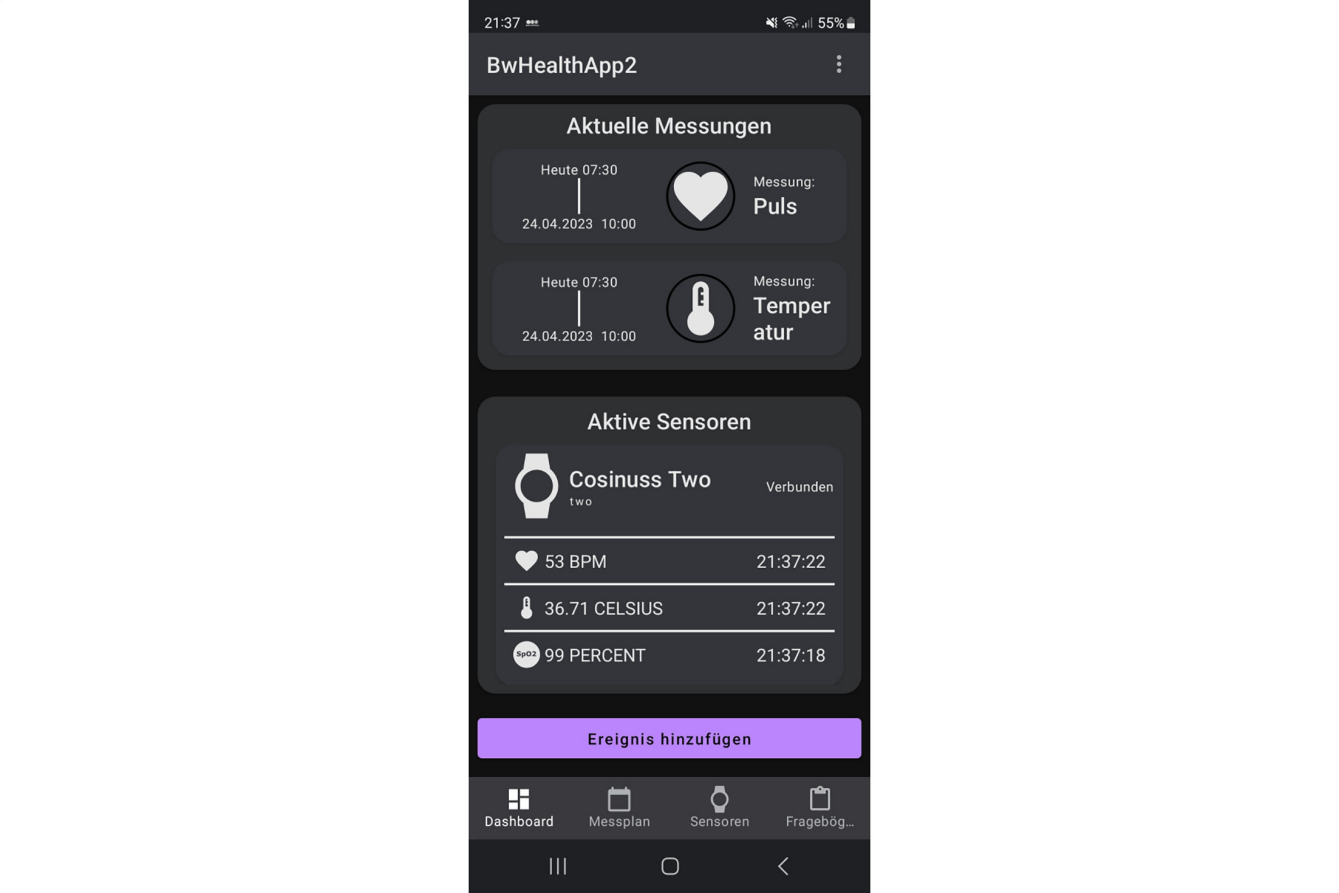
User interface of the bwHealthApp dashboard when using the in-ear wearable during the pilot study.

Outpatients were asked to use the bwHealthApp system as much as possible to evaluate the use in everyday life between therapy sessions. The app could be accessed with anonymized login credentials, starting at the dashboard (Figure 2). The app sent push notifications as reminders to complete the ePROs and use the wearables. Through bwHealthApp, health status was assessed daily using 10 items, whereas quality of life was assessed weekly using 30 items. At the same time, commercial wearables for the continuous measurement of activity and vital signs could be connected to bwHealthApp via Bluetooth Low Energy. bwHealthApp saved the values on the smartphone and sent them to a server when connected to the internet, as previously described [12]. The data could be viewed almost in real time, with a delay of only a few minutes.

User-friendliness, satisfaction, and acceptance of bwHealthApp were assessed using a paper-and-pencil survey at the beginning and end of the study. In the intervening period, a short interview was conducted. These sessions were scheduled at the same time as the patients’ respective treatment sessions at the clinic, resulting in different schedules due to various therapy regimens. The midpoint interview was conducted 2-4 weeks after the initial survey, and the final survey was conducted 4-8 weeks after the initial survey. Moreover, digital competence was assessed at the beginning of the study. The Technology Usage Inventory (TUI) [13] was completed before and after using bwHealthApp.

### Outcomes

#### Adherence

Prior to using the bwHealthApp system, the expected self-perceived use rate was evaluated on a 6-point scale (1=“not weekly” to 6=“continuously daily”), as well as use scenarios (sleep, everyday activities, physical activities, illness, others; multiple answers possible). At the end of the study, the same scales were used to assess the use rate. Objectively, adherence was assessed on the basis of the actual use of the wearables or ePROs sent. The use rate was calculated as the proportion of days of bwHealthApp use during the study period. Adherence to digital systems depends on whether users can understand and operate them. Digital skills and previous experience, which play a key role in adherence, were assessed to characterize the sample. At the beginning of the study, knowledge of and previous experience with wearables was examined. Digital competence was assessed on the basis of the Digital Competence (DigiKom) questionnaire [14] using the domains of knowledge and skills [15]. Participants were asked whether they had access to smartphones, computers, and tablets; how often the devices were used; and how they rated the handling of the devices. To assess the use of digital devices, the participants were asked to rate 8 statements on a 5-point Likert scale (1=“I do not agree at all” to 5=“I fully agree”).

#### Acceptance and Satisfaction

At the beginning and end of the study, satisfaction, convenience of use, and wearing comfort were assessed on a 5-point Likert scale (1=“I do not agree at all” to 5=“I fully agree”). Technology-specific and psychological factors—important for the acceptance of new technologies—were examined using the TUI [13], which assesses curiosity, anxiety, interest, user-friendliness, usability, and skepticism on a 7-point scale. The TUI also measures the intention to use.

#### Wearable Data

Each participant was equipped with a commercially released wearable: the Beurer AS 99 Pulse wristband or the Cosinuss Two in-ear wearable. The selection of wearables was based on the price range and the location of the manufacturers in Germany, which simplified communication with the manufacturers for wearable–app integration. Furthermore, both wearables operate according to open Bluetooth standards, which allow data to be accessible without the data being sent to a manufacturer’s server. The wrist wearable was worn on the nondominant hand and measured heart rate; sleep duration (manually derived); and activity parameters such as daily step count, distance, activity time, and calories burned. The in-ear sensor measured heart rate, body temperature, and oxygen saturation.

### Data Processing and Analyses

The data collected via bwHealthApp could be visualized and exported via a web client. These data were used to calculate the number of days on which wearables and ePROs were used. Vital measures outside the physiologically feasible range were excluded from the analysis. The proportion of excluded data was used as a data quality measure. The included values were as follows: heart rate 35-250 beats per minute, temperature 34-42 °C, and oxygen saturation above 84%. The file showed the remaining raw data and daily average values of the vital parameters as well as daily sum values of the activity parameters. Paper-and-pencil surveys were digitized and analyzed in SPSS (version 28; IBM Corp). Missing values were excluded from analysis. Most of the presented statistics are descriptive and were used to assess general feasibility. After ensuring that the statistical assumptions were met, between-group *t* tests (2-tailed) were performed for independent groups (wrist and in-ear wearables).

### Ethical Considerations

Ethical approval for the pilot study was obtained from the University of Tübingen Ethics Committee (046/2022BO1). All participants provided informed consent. The data reported are anonymized. The participants were not compensated for their participation.

## Results

### Study Sample

**Figure 3.**
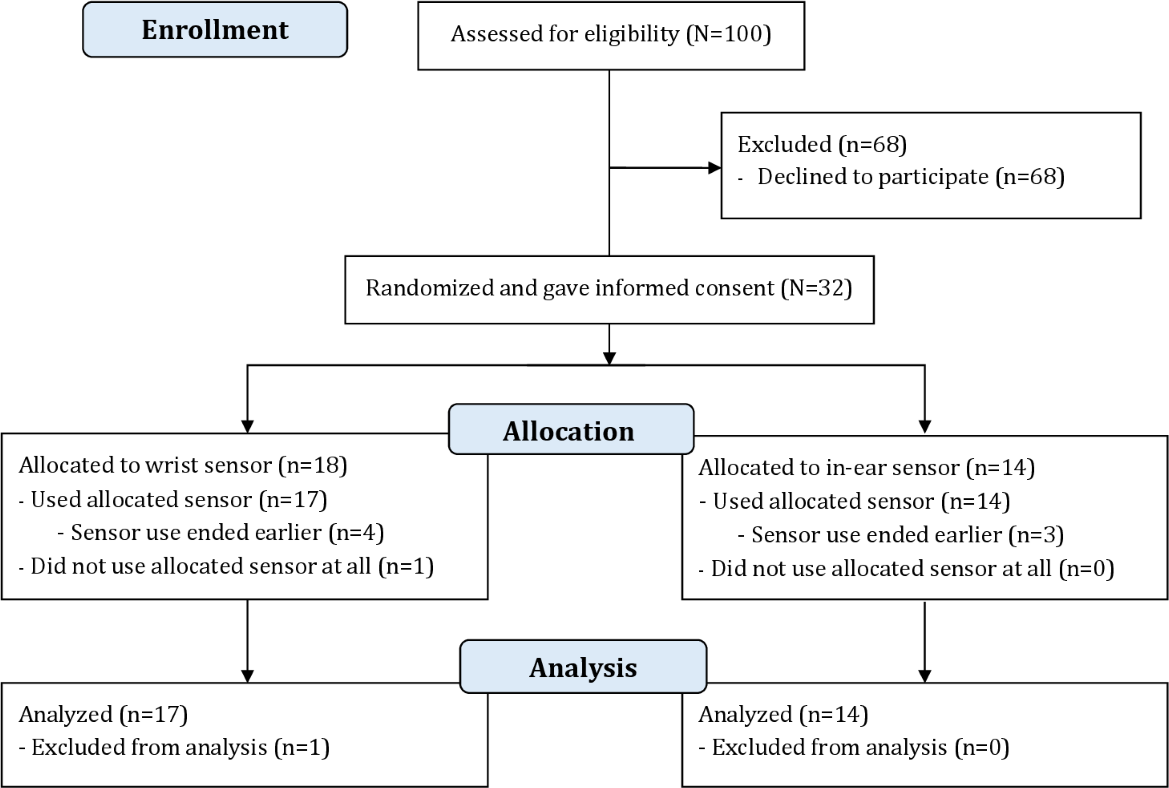
Patient flowchart.

As Figure 3 shows, 32 outpatients participated in the study between July and November 2022 (17/31, 55% male; mean age 59.3 y, range 39‐74 y). One patient did not record data and was excluded from analysis. Of the remaining 31 outpatients, 6 ended data collection earlier due to handling issues with the provided phone (n=4) or disease progression (n=2). Additionally, 1 outpatient did not complete the final paper-based questionnaire. In total, 17 patients received the wrist wearable, whereas 14 used the in-ear wearable (Table 1). Of the 31 patients, 10 (32%) were being treated for esophageal or gastric cancer, 9 (29%) for colorectal cancer, 6 (19%) for biliary cancer, 4 (13%) for pancreatic cancer, and 2 (7%) for liver cancer. In 29 of 31 patients, the primary tumor had already metastasized. In total, 17 types of treatment strategies were used: of the 31 participants, 23 (74%) were being treated with systemic chemotherapeutic agents as monotherapy or combination therapy, 11 (35%) with targeted therapy, and 12 (39%) with immunotherapy.

**Table 1.** Characteristics of study participants (N=31).

Characteristic	Wrist wearable users (n=17)	In-ear wearable users (n=14)
Age (years), mean (SD)	56.5 (10.3)	62.6 (7.9)
Sex, n (%)		
Male	10 (58.8)	7 (50)
Female	7 (41.2)	7 (50)
Smartphone used, n (%)		
Own smartphone	10 (58.8)	9 (64.3)
Study smartphone	7 (41.2)	5 (35.7)
Cancer type, n (%)		
Esophageal/gastric	6 (35.5)	4 (28.6)
Colorectal	5 (29.4)	4 (28.6)
Biliary	3 (17.6)	3 (21.4)
Pancreatic	2 (11.8)	2 (14.3)
Liver	1 (5.9)	1 (7.1)
Treatment intent, n (%)		
Curative	1 (5.9)	1 (7.1)
Palliative	16 (94.1)	13 (92.9)
Therapy type^a^, n (%)		
Systemic chemotherapy	11 (64.7)	12 (85.7)
Targeted therapy	7 (41.2)	4 (28.6)
Immunotherapy	7 (41.2)	5 (35.7)

a As either monotherapy or combination therapy.

### Adherence

Study participation averaged 31 (SD 10.4) days. ePROs were sent on 46% (449/971) of the days, and the wearable was connected on 61% (593/971) of the days [16]. Participants reported a total of 571 ePROs in bwHealthApp, with an average of 18 (SD 14) ePROs per participant.

At the beginning of the study, 73% (23/31) of the participants stated that they intended to use bwHealthApp daily. At the end of the study, 54% (16/30) reported daily use of bwHealthApp. In fact, 17% (5/31) of the participants used the bwHealthApp system every day.

During the analysis, an explorative finding indicated that on the basis of use behavior, the participants could be divided into heavy (n=18) and light users (n=13)—participants who used the app for more and less than 50% of the study duration, respectively. Notably, 77% (10/13) of light users used a study smartphone, whereas for heavy users, this percentage was 11% (2/18).

The participants’ average digital competence score based on their self-reported skills was 30.78 (SD 11.19) points, where the maximum achievable score was 40. Notably, 20 participants were already familiar with wearables, mostly from their own environment (13/20, 65%). Eleven patients already used a wearable in their daily lives. The main reason given for not having used wearables previously was the lack of need. In terms of technology acceptance, the bwHealthApp system was rated highest on the scales of usefulness (mean 7.78, SD 1.59) and curiosity (mean 6.30, SD 1.93). Skepticism toward the new technology was low (mean 3.77, SD 1.94).

### Acceptance and Satisfaction

**Figure 4.**
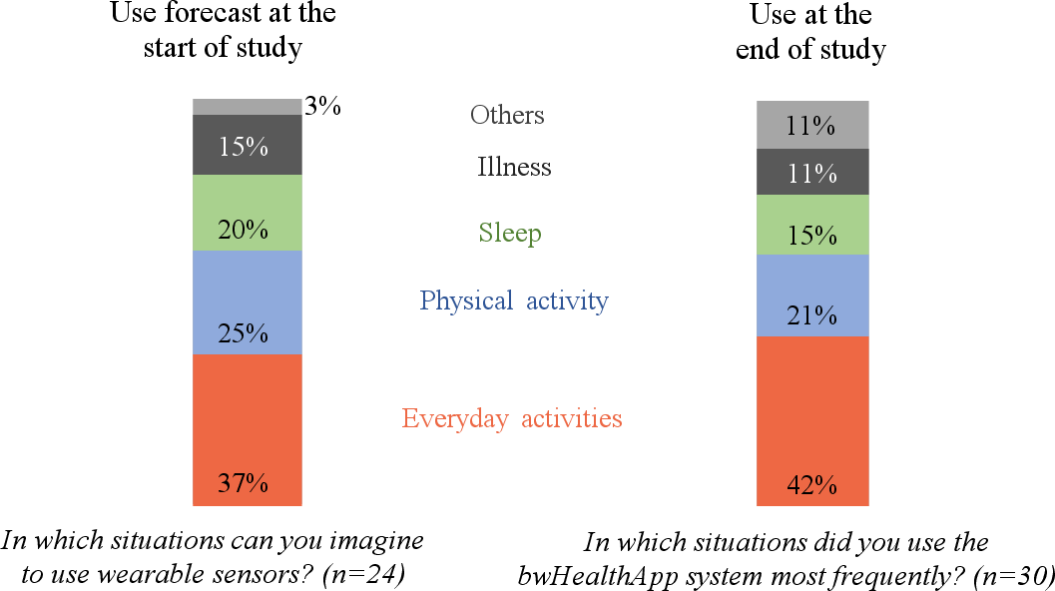
Preferred situations for using bwHealthApp that were forecast at the beginning and reported at the end of the study.

At the end of the study, 60% (18/30) of the participants were satisfied with bwHealthApp. Moreover, 70% (21/30) reported that they were willing to continue using the app. No participant rated the use of wearables for medical purposes as unsuitable, and 70% (21/30) rated the system as easy to use. bwHealthApp was most frequently used during everyday and physical activities (Figure 4).

### Wearable Data

#### Data Quantity and Quality

**Figure 5.**
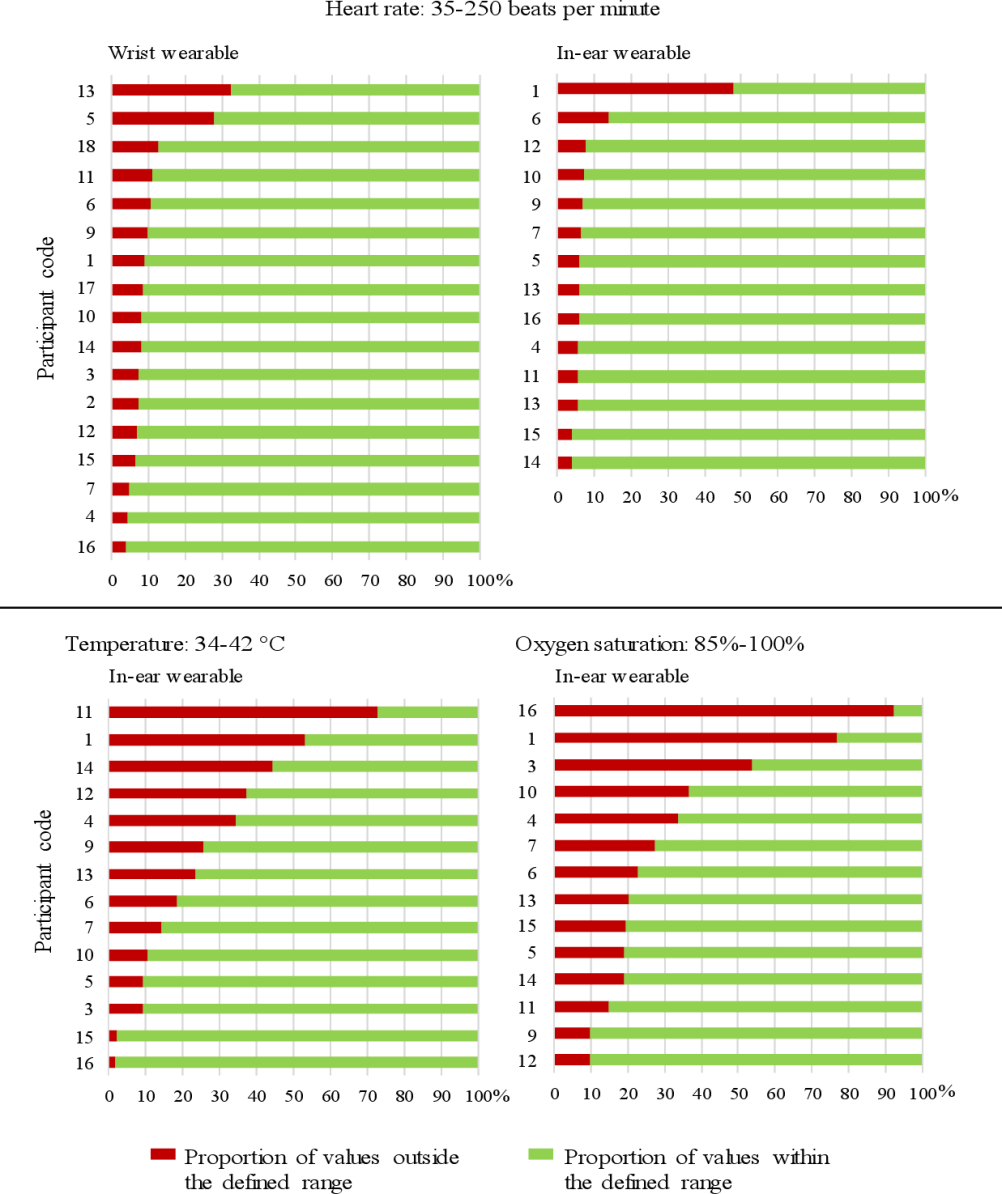
Visualization of the proportion of excluded values of heart rate (wrist-, in-ear wearable) as well as temperature and oxygen saturation (in-ear wearable) per participant. Heart rate measurement from wrist wearable and in-ear wearable; temperature and oxygen saturation measurements from in-ear wearable only.

Of 20,067,891 wearable measure points, an average of 543,528 (SD 462,028) heart rate data points and 1725 (SD 1109) activity parameter data points were collected per participant. For the in-ear sensor, the average number of data points collected for heart rate, temperature, and oxygen saturation was 327,559 (SD 450,404), 355,764 (SD 476,509), and 88,005 (SD 13,631), respectively. On average, 11% (SD 8%, range 4%-33%, wrist) and 10% (SD 11%, range 4%-48%, in-ear) of the heart rate values were outside the physiological range and were considered nonsense data (Figure 5). Notably, 29% (SD 22%, range 3%-76%) and 33% (SD 25%, range 10%-92%) of the data points for temperature and oxygen saturation, respectively, were excluded. According to bwHealthApp data, the nightly sleep duration ranged from 1 to 755 minutes; the median (IQR) was 255 (36-399) minutes, corresponding to 4 hours of sleep. The sleep function was activated on 139 of the total 380 days on which the wrist wearable was used by all participants.

#### Wearable Differences

Table 2 and Table 3 show the results of the analysis of differences between the two wearable groups. The expected use rates reported at the beginning of the study differed significantly from the postuse self-assessment depending on wearable type (Table 2). Wrist wearable users (preuse: mean 5.43, SD 0.85; postuse: mean 4.86, SD 1.35) reported more frequent use than in-ear wearable users (preuse: mean 3.54, SD 1.71; postuse: mean 3.23, SD 1.79). Objectively, bwHealthApp use (in days), in terms of wearable use and ePRO completion did not differ significantly between the two groups (Table 3). Descriptively, the use of the wristband was higher. The wrist and in-ear wearables differed significantly in wearing comfort, with a higher rating for the wristband. Overall satisfaction showed no significant difference between the two wearable groups.

**Table 2.** Results of the repeated measures ANOVA with within-subjects factors to test for differences between the wrist and in-ear wearable groups for self-perceived use rates at the beginning and end of the study. Self-perceived use rates were based on a scale of 1 (lowest) to 6 (highest) at various measurement time points (beginning and end of the study) for each wearable group (wrist or in-ear wearable).

Effect	*F* test (*df*)	*P* value^a^	Partial η²
Measurement time point (n=27)^b^	2.03 (1)	.17	.08
Wearable group (n=27)^b^	13.97 (1)	<.001	.36
Measurement time point × wearable group (n=27)^b^	0.18 (1)	.67	.01

a 2-sided *P* value.

b 4 missing values.

**Table 3.** Results of the *t* test for independent samples to examine differences between the wrist and in-ear wearable groups (N=31); satisfaction and wearing comfort were scored on a scale of 1 (lowest) to 5 (highest), and intention to use was scored on a scale of 0 (lowest) to 200 (highest).

	Wrist wearable (n=17), mean (SD)	In-ear wearable (n=14), mean (SD)	*t* test (*df*)	*P* value^a^	Cohen *d*
ePRO^b^ use (days)	15.47 (9.21)	10.57 (8.53)	1.52 (29)	.14	0.55
Wearable use (days)	20.94 (10.08)	14.21 (10.59)	1.81 (29)	.08	0.65
Satisfaction^c^	3.50 (1.30)	3.64 (1.22)	−0.35 (28)	.73	−0.13
Wearing comfort^c^	4.13 (0.62)	3.21 (1.31)	−11.17 (28)	.03	0.91
Intention to use	120.24 (50.71)	120.93 (58.93)	−0.04 (29)	.97	−0.01

a 2-sided *P* value.

b ePRO: electronic patient-reported outcome.

c 1 missing value (n=16).

## Discussion

### Principal Findings

This pilot study examined the feasibility of an RPM system (bwHealthApp) incorporating ePROs and two types of wearables (wrist and in-ear) for outpatients with gastrointestinal cancer undergoing systemic therapy. A heterogeneous sample of patients, including dropouts, showed an adherence rate of 61%. Descriptively, perceived use was higher than the actual use. The system was primarily used during everyday activities. One possible influencing factor on the frequency of use of the app could be the use of one’s own smartphone or an additional one. Despite the overall high digital competence, the dropout rate was descriptively higher among participants using a study smartphone. Nevertheless, most participants were satisfied with bwHealthApp and were willing to continue using this RPM system. Participants exhibited low skepticism about this new technology. The use of bwHealthApp by the participants enabled the collection of hundreds of ePROs and millions of wearable data points.

The two wearables showed differences. On average, one-third of the in-ear temperature and oxygen saturation values were outside the physiological range. In contrast, this proportion was approximately 10% for heart rate. The sleep function of the wrist wearable was activated on fewer than half of the days it was used. Overall, the wristband performed better than the in-ear wearable. The results showed subjectively and descriptively better adherence and a significantly higher wearing comfort.

### Comparison to Prior Work

The findings of this study contribute to the growing field of RPM. It emphasizes the significance of technical solutions and their assessment in feasibility studies, with the vision of enhancing outpatient cancer therapy.

Patients with advanced cancer and a mean age of 59 years demonstrated a high level of digital competence and acceptance of technology. This finding lends further credence to the prevailing assumption that individuals older than 55 years seldom encounter challenges in using wearable technology [17]. The participants used bwHealthApp on average on 60% of the study days [16]. This level of adherence is comparable with previous RPM studies [18-20]. However, given that as many as 50% of participants were excluded from previous studies, adherence to bwHealthApp may be higher [10,21]. The observed use rate was lower than that estimated by the participants at the beginning of the study. One potential explanation for this phenomenon is that users overestimated themselves, as has been reported for physical activity [22]. The app was assessed as a beneficial tool, a conclusion that is corroborated by previous findings in related oncology disciplines [23,24]. The accuracy of the health data was not assured, especially for the in-ear sensor. A considerable percentage of body temperature and oxygen saturation values was found to be outside the physiological range. This suggests that, in contrast to previous studies, a field survey of biosignals in the ear canal may not be feasible [25]. It is not clear if medical devices would be a more reliable source of data in this case. It is generally assumed that an accurate measurement, particularly of heart rate and step count, is feasible with other forms of wearable technology [26-29]. Despite the necessity of filtering the heart rate values, the proportion of excluded data points was consistent with the range observed with other wearables that have been reported to achieve acceptable measurement accuracy [30]. The better performance of the wrist wearable may provide a rationale for the widespread use of such devices within RPM systems [31,32].

### Strengths and Limitations

The study protocol was executed in accordance with existing treatment regimens. However, the integration of the study into the the participants’ treatment plans resulted in varying study periods and clinical measurement intervals. This may have influenced the evaluation of the bwHealthApp system. However, these issues also arise when bwHealthApp is used routinely, as data cannot be collected from all patients at the same time. In addition, the study period was limited to a few therapy cycles. However, treatments often last for years. Future research should evaluate the use of the app over a longer period.

The heterogeneous sample of patients with advanced cancer met the study’s objective of obtaining feedback on the system from a wide array of users. However, it is important to note that influencing factors cannot be kept constant in this approach, and the generalizability of the findings is limited. A potential limitation to consider is the possibility that the study participants were predominantly technophiles, potentially skewing the results toward individuals with higher digital competence. In future studies, a sample with a similar treatment regimen should be investigated, if possible with a higher degree of personalized therapy. Furthermore, an analysis of correlations between the bwHealthApp data and clinical data may provide insights for improving treatment benefit.

During the study, participants who were non-Android users were provided with Android smartphones, which may have affected the frequency of use and the evaluation of user-friendliness and acceptance of bwHealthApp. The development of an iOS version of bwHealthApp is planned. Furthermore, adherence may have been underreported in the Results section. Despite wearable use, functionality errors in bwHealthApp may result in the absence of data recording, which may be attributed to factors such as Bluetooth deactivation. In the future, it will be necessary to determine whether other wearables offer superior functionality. Further analysis of factors influencing wearable use is necessary, as the high standard deviation suggests interindividual differences.

In certain instances, validated questionnaires were not used due to their extensive number of items. The implementation of daily questionnaires and in-clinic surveys presented a potential challenge, as it could potentially overwhelm patients, leading to a decline in response rate or changes in response behavior. Nevertheless, the data collected in this pilot study provide validation and a baseline for the scales in future studies.

The technology used in this study is not considered a form of medical device. To interpret the reported values, the patients need to be examined by medical staff. As these values are displayed in bwHealthApp, the limited informative value of the single measures needs to be communicated to participants. Regarding the wearable-reported values, the method by which these are calculated from the sensor raw data remains unclear. For enhanced research quality, it is crucial that the manufacturers provide comprehensive and transparent information. In a related manner, all Bluetooth-supported wearable devices should possess the capability to query the data via a system such as bwHealthApp.

Sleep duration was manually configured on the wrist-wearable device. The use of a sensor model capable of determining sleep would be an advancement. The implementation of the in-ear wearable was likely hindered by the challenges posed by the COVID-19 pandemic. The use of a face mask may have contributed to a decline in comfort levels and adherence, which may have contributed to the observed interindividual variability in the quality of values measured by the in-ear wearable.

Despite its limitations, this pilot study makes a substantial contribution to the development of bwHealthApp in the field of personalized oncology.

### Future Directions

The results highlight the importance of conducting feasibility studies with new RPM systems. Due to the rapid pace of research and development in this field, it is necessary to continuously develop RPM systems. bwHealthApp offers the possibility of connecting additional wearables. Classic wearables with an extension of biosignals are one option. However, implantable sensors in the port or tumor tissue, which do not require adherence to device use, are also viable options, as gastrointestinal tumors, in particular, are easily accessible by endoscopy. In any case, close cooperation between the medical and technical informatics disciplines is required. This also applies to data analysis, as there are millions of data points available. The use of artificial intelligence should be considered. Future research should analyze the data collected from a homogeneous patient group in a long-term study and examine their correlations with clinical data such as staging data and laboratory values, with the aim of improving outpatient cancer therapy. In this context, future studies should also investigate the clinical process integration and assess the acceptance of RPM by medical staff.

### Conclusions

This pilot study on the bwHealthApp system demonstrates the feasibility and acceptance of continuous monitoring via a mobile app and wearables during outpatients’ systemic cancer therapy, including targeted therapies. Patients with advanced cancer were able to use the RPM system. They found it acceptable and were generally satisfied with bwHealthApp. The wearable type was an important aspect due to differences between the two devices. This emphasizes the need for further technological development and integration through a flexible system such as the bwHealthApp system.

The collected RWD can be used to further personalize the therapy and care of patients with cancer. The next step is to analyze the app data and clinical data using artificial intelligence. With sufficient data, predictors may be developed to support an automated alert system, offering new possibilities for outpatient cancer treatment.

## Abbreviations

ePRO: electronic patient-reported outcome
RPM: remote patient monitoring
RWD: real-world data
TUI: Technology Usage Inventory
